# Ten-Year Single-Center Study Examining Patient Survival after Commencing Long-Term Peritoneal Dialysis

**DOI:** 10.3390/jcm12237283

**Published:** 2023-11-24

**Authors:** Jesús Venegas-Ramírez, Karla Esmeralda Barrios-Mora, Eder Fernando Ríos-Bracamontes, José Guzmán-Esquivel, Martha Irazema Cárdenas-Rojas, Efrén Murillo-Zamora

**Affiliations:** 1 Departamento de Nefrología, Hospital General de Zona No. 1, Instituto Mexicano del Seguro Social, Av. Lapislázuli 250, Col. El Haya, Villa de Álvarez 28984, Mexico; jvenegas4@ucol.mx; 2 Departamento de Medicina Interna, Hospital General de Zona No. 1, Instituto Mexicano del Seguro Social, Av. Lapislázuli 250, Col. El Haya, Villa de Álvarez 28984, Mexicoeder.rios@imss.gob.mx (E.F.R.-B.); 3 Unidad de Investigación en Epidemiología Clínica, Instituto Mexicano del Seguro Social, Av. Lapislázuli 250, Col. El Haya, Villa de Álvarez 28984, Mexico

**Keywords:** kidney failure, chronic, peritoneal dialysis, survival rate, treatment outcome

## Abstract

In patients with end-stage kidney disease (ESKD), peritoneal dialysis (PD) is increasingly being adopted in developing nations due to its cost-effectiveness and advantages as a home-based treatment. However, the survival outcomes of chronic PD patients in Mexico, where the burden of ESKD is high, remain poorly understood. This study aimed to assess the survival experience of long-term PD patients and evaluate its determinants. A single-center cohort study collected clinical and epidemiological data for long-term PD initiations between February 2013 and January 2023. The analysis, which utilized Kaplan–Meier and Cox proportional hazard regression methods, included data from 370 patients. The overall mortality rate was 4.7 per 1000 person-months, with a significant decrease in survival rates observed between months 24 and 36 post-PD initiation. Older age at PD initiation and the use of continuous ambulatory peritoneal dialysis, as compared to the automated modality, were associated with an increased risk of mortality. The study provides valuable insights into the survival outcomes of chronic PD patients in Mexico and suggests the need to optimize treatment strategies to enhance long-term prognoses for patients with ESKD. Further research is needed to better understand the factors influencing survival in this population.

## 1. Introduction

Renal replacement therapy (RRT) is a life-sustaining treatment for patients with end-stage kidney disease (ESKD) who experience a progressive decline in kidney function. The initiation of RRT, whether through peritoneal dialysis (PD), hemodialysis, or renal transplantation, marks a crucial milestone in the management of ESKD [1].

In developed nations, hemodialysis is predominantly employed as the first choice to start RRT, whereas PD has been progressively adopted in developing nations [2]. Chronic PD presents several advantages, such as reduced expenses, the ability to administer treatment at home, and the use of a single access point, among others [3]. The two main modalities of long-term PD are continuous ambulatory peritoneal dialysis (CAPD) and automated peritoneal dialysis (APD).

Assessing the survival outcomes of patients undergoing chronic PD is crucial for informing clinical decision making and enabling healthcare professionals to evaluate the efficacy of various treatment approaches and identify areas for improvement. However, published survival rates have shown heterogeneity, with most studies focusing on Asian or European populations [4,5,6,7,8,9,10]. In Mexico, there is a scarcity of published data on this matter [11,12,13,14], particularly recent studies, which may be relevant considering the observed upward trend in ESKD-related burden over the past decade [15].

Therefore, the objective of this study was to evaluate the survival experience of patients who initiated RRT using any long-term PD modality. By analyzing a cohort of patients and considering clinical and demographic variables, we aimed to elucidate the factors associated with improved or diminished survival outcomes in this population. Understanding the determinants of survival in patients undergoing RRT can provide valuable guidance to healthcare providers in optimizing treatment strategies, enhancing patient care, and ultimately improving the long-term prognosis for individuals with ESKD.

## 2. Materials and Methods

We conducted a cohort study in the first half of 2023 at the PD unit of a public hospital affiliated with the Mexican Institute of Social Security (IMSS, the Spanish acronym). The hospital is situated in the west-central region of the country, specifically in the state of Colima. The study employed a mixed temporality design and included patients who initiated long-term RRT via PD between February 2013 and January 2023 at the participating healthcare facility.

Individuals aged 18 years or older, at the initiation of long-term PD, were considered potentially eligible for the study and were identified using the records from the dialysis unit where the study took place. Patients with missing clinical or epidemiological data of interest were excluded. Relevant clinical and epidemiological data were collected from medical records and death certificates, if available.

The analysis focused on the survival time, which was measured as the duration (in months) between the date of the first PD session (starting event) and the date of death (failure event) from any cause. The censoring variable included patients who did not experience the event (i.e., did not die) during the follow-up period. The study end date was 30 June 2023, and it was used to calculate the time at risk for these participants.

Patients whose follow-up was discontinued due to administrative reasons (e.g., loss of healthcare service rights at the institution, migration, or treatment abandonment), change in PD modality due to peritonitis, or those who underwent renal transplantation were also censored. In these cases, the time at risk was determined using the date of the last medical consultation, the date of initiation of another dialysis modality, or the date of transplant surgery, respectively.

Survivor functions and 95% confidence intervals (CIs) were computed using the Kaplan–Meier method. Factors associated with the risk of death were assessed using hazard ratios (HRs), and a multivariate Cox proportional hazard regression model was employed. The Local Committee in Health Research (601) of the IMSS granted approval for the study (R-2023-601-017).

## 3. Results

Data from 370 patients were analyzed, resulting in a total follow-up period of 15,646 person-months. Most participants (66.8%) received CAPD, and approximately 6 out of 10 enrolled patients were male (63.5%). The median age (and total range) in the study sample was 59.9 (18.3–89.4) years at the start of long-term PD. Other characteristics of the study sample, based on the failure event outcome, are summarized in Table 1.

When identifying the pathology potentially leading to the onset of ESKD, type 2 diabetes mellitus was the most frequent, identified in nearly three-quarters of participants (74.1%). Arterial hypertension was the second leading disease related to the progression of renal failure (14.6%), while the remaining fraction was attributed to other illnesses.

A total of 50 peritonitis cases were observed. The median interval from PD initiation to the onset of peritonitis was 56.5 months, ranging from 19.9 to 158.1 months. When compared to participants who did not present peritoneal complications during the follow-up, those with peritonitis were younger (*p* = 0.001), with median ages of 60.7 and 53.6 years, respectively. 

A low frequency of renal transplantation was documented, occurring in only six patients. Therefore, the overall frequency in the study sample was 1.4%. The median time on RRT in this group of patients was 46.2 months, with a total range of 26.2–88.7 months. Generally, these patients were young, with a median age estimate of 32.2 (27.4–41.2) years at the start of PD. 

**Figure 1:**
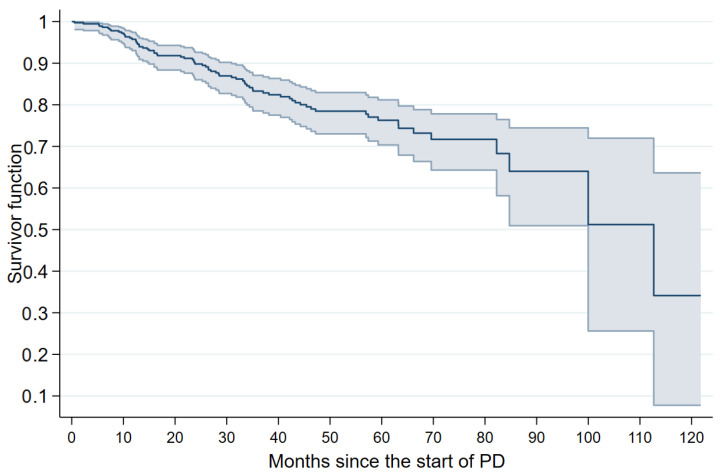
Kaplan–Meir curve of adults initiating renal replacement therapy via peritoneal dialysis (PD), Mexico 2013–2023. Note: The survivor functions and 95% confidence intervals are presented.

We observed a total of 73 deaths, resulting in an overall mortality rate of 4.7 per 1000 person-months. The mortality rates, stratified by modality, were 3.1 and 5.7 in APD and CAPD, respectively. Figure 1 illustrates the computed overall survivor functions.

As presented in Table 2, the survival rates within the first two years of treatment were approximately 90% or above. The most significant decrease in the computed functions was documented from month 24 to 36 since the initiation of PD, where the rates decreased from 89.7% (95% CI 85.8–92.5%) to 83.2% (95% CI 78.5–87.0%). At 72 months, the second highest decrease occurred compared to 60 months, with rates of 71.9% (95% CI 64.6–77.9%) and 76.3% (95% CI 70.4–81.2%), respectively.

In the multiple Cox proportional hazards regression model (Table 3), it was observed that patients who were older at the initiation of PD exhibited lower survival rates. For each additional year of age, the risk of mortality increased by approximately 2% (HR = 1.02, 95% CI 1.01–1.03; *p* = 0.040). Additionally, irrespective of the selected cut-off, CAPD patients demonstrated an 89% higher risk of mortality compared to APD patients (HR = 1.89, 95% CI 1.08–3.01; *p* = 0.026). Neither the gender nor the year of PD initiation was significantly associated with the outcome of interest. However, they were kept in the multiple model to include unobserved variables that might affect the survival rates (e.g., gender-related risk behaviors or improvements in provided interventions).

## 4. Discussion

In this study, we investigated the survival experience of patients undergoing long-term PD and identified factors associated with survival outcomes. Our findings offer valuable insights into the characteristics and prognosis of PD patients in Mexico. However, it is essential for readers to consider that our results are based on the experience of a single center when interpreting the findings.

The survival rates within the first two years of treatment were generally favorable, with rates above 90%. However, a notable decline in survival rates was observed between months 24 and 36 after the initiation of PD, with rates decreasing from 89.7% to 83.2%. Another significant decrease occurred at 72 months compared to 60 months, with rates of 71.9% and 76.3%, respectively.

Heterogenous survival rates have been published, but our overall estimates are similar to those computed in Japanese [7] and Korean [8] populations. They are also higher than those from Spain [5], Turkey [4], China [6], Peru [9], and Indonesia [10]. The survival rates of long-term PD patients can vary across nations due to differences in healthcare infrastructure, access to medical resources, socioeconomic factors, healthcare policies, and population health characteristics [16]. Addressing these factors and implementing evidence-based strategies can help improve patient outcomes and narrow the survival rate gap between different nations. The dialysis unit where the study was conducted belongs to the IMSS, a leading health institution at the national level, providing RRT [17]. High-quality medical and social services are provided at this institution.

Our multiple Cox proportional hazards regression model demonstrated that older age at PD initiation was associated with lower survival rates. With each additional year of age, the risk of mortality increased by approximately 2%. Older patients may experience reduced physiological reserves and compromised organ function [18], rendering them more susceptible to the stresses of PD treatment. Additionally, the prevalence of comorbid conditions may be higher in older patients compared to younger ones. These factors may lead to challenges in adhering to PD treatment regimens, resulting in suboptimal therapy and poorer outcomes [19]. Collectively, these factors may have contributed, at least partially, to the observed scenario.

Furthermore, regardless of the selected cut-off, CAPD patients exhibited an 89% higher risk of mortality compared to APD patients. These findings align with recently published data [20]. The observed scenario might be related to various factors and one of these might be that the manual nature of CAPD may lead to variations in the adequacy of dialysis and the clearance of waste products and toxins [21], which can impact overall health and survival. In addition, the frequent exchanges in CAPD can cause fluctuations in fluid and electrolyte levels [22,23], potentially leading to imbalances and associated complications. Finally, APD may provide greater convenience and ease of adherence, ensuring more consistent and regular dialysis sessions [24].

In the context of long-term PD modalities, peritonitis risk remains a critical concern [25]. Recent studies have revealed variations in peritonitis incidence between different modalities [26,27]. While prior literature has suggested a potential reduction in peritonitis risk in APD patients, our research uncovered a statistically significant difference, with 56.0% of cases occurring in APD compared to 44.0% in CAPD (*p* < 0.001). The observed variation prompts further inquiry into factors that may contribute to these differences, including patient demographics, adherence to aseptic techniques, and the potential impact of the automated nature of APD [28,29]. Understanding and addressing these nuances in peritonitis risk across peritoneal dialysis modalities is crucial for optimizing patient care and outcomes in the long-term management of end-stage renal disease.

Type 2 diabetes mellitus was identified as the most common pathology leading to ESKD, observed in nearly three-quarters of the participants (74.1%). Arterial hypertension was the second leading cause, accounting for 14.6% of cases, while the remaining fraction was attributed to other illnesses. This pattern aligns with the high prevalence of diabetes and hypertension and diabetes in Mexico [14], which significantly contribute to the burden of ESKD in the country.

Renal transplantation was infrequent, documented in only six patients, resulting in an overall frequency of 1.4% in the study sample. In Mexico, like in many other countries, there is a significant shortage of organ donors, posing a major challenge to the effective treatment of patients with end-stage organ failure. This scarcity of donors is multifactorial, influenced by cultural beliefs, religious perspectives, inadequate awareness campaigns, and mistrust in the medical system [30,31,32]. In Mexico, the rates of organ donors are low [33], and by the end of the first bimester of 2023, more than 22 thousand people were waiting for an organ donation, with most of them in need of a kidney or cornea [34].

One significant limitation of our study is that it was conducted at a single center within a specific healthcare institution, potentially limiting the generalizability of our findings to some extent. However, it is important to note that the IMSS, being a part of the Mexican public healthcare system and providing services to approximately 68% (83.2 million people) of the total population of the country, serves a diverse and heterogeneous population. Despite the restriction to a single center, the inclusion of patients from IMSS provides valuable insights into the healthcare experiences and outcomes of a substantial segment of the population. In future research, conducting multicenter studies could enhance the generalizability of our findings and lead to a more comprehensive understanding of the topic under investigation.

Another limitation of this study that must be highlighted is the limited number of covariates that were evaluated, which constrains a deeper understanding of the factors affecting the outcomes under investigation. Mexico, like many other countries, is currently witnessing an increase in the utilization of renal replacement therapy, primarily attributed to the pandemic of type 2 diabetes mellitus [35]. Our study represents an initial step in addressing this issue, given the limited availability of published data within this specific population. Subsequent studies must assess a larger number of patient characteristics to provide a broader framework for determining the survival of patients undergoing long-term peritoneal dialysis.

## 5. Conclusions

The findings of this study highlight the importance of considering age and PD modality when evaluating survival outcomes in patients with ESKD undergoing long-term PD. Older age at PD initiation and the use of CAPD were associated with reduced survival rates. These results have implications for healthcare providers in optimizing treatment strategies and improving the long-term prognosis for ESKD patients. Further research is needed to better understand the factors influencing survival in this population and to validate these findings in larger and more diverse cohorts.

## Figures and Tables

**Table 1:** Characteristics of the study sample for selected variables according to the failure event status, Mexico 2013–2023.

	Failure Event (Fatal Outcome)		*p*	**Person-Time**
	No (*n* = 297)	Yes (*n* = 73)		
Gender				
Female	107 (36.0)	28 (38.4)	0.711	5269
Male	190 (64.0)	45 (61.6)		10,377
Age (years)	59.5 (18.3–89.4)	62.1 (28.7–78.6)	0.207	15,646
Subjacent cause of ESKD				
T2DM	219 (73.7)	55 (75.4)	0.812	10,741
Hypertension	45 (15.2)	9 (12.3)		2436
Other	33 (11.1)	9 (12.3)		2469
Treatment modality				
APD	104 (35.0)	19 (26.0)	0.144	6225
CAPD	193 (65.0)	54 (74.0)		9422

Abbreviations: T2DM, type 2 diabetes mellitus; ESKD, end-stage kidney disease; APD, automated peritoneal dialysis; CAPD, continuous ambulatory peritoneal dialysis. Notes: (1) The absolute frequencies (*n*) and relative frequencies (%) are presented for all variables except the Age variable, for which the median and total range are provided instead. (2) The *p*-values from chi-squared or Mann–Whitney tests are provided as appropriate. (3) The person-time was measured in months (person-months).

**Table 2:** Survivor functions in the study sample, Mexico 2013–2023.

*t* (Months)	Patients at Risk (*n*)	Failures (*n*)	Survivor Function (95% CI)
1	372	1	99.7 (98.1–99.9)
3	371	4	99.5 (97.9–99.8)
6	365	10	98.4 (96.5–99.3)
12	325	13	95.5 (92.8–97.2)
18	300	6	91.6 (88.2–94.1)
24	267	17	89.7 (85.8–92.5)
36	194	10	83.2 (78.5–87.0)
48	144	3	78.4 (73.0–82.9)
60	96	4	76.3 (70.4–81.2)
72	39	1	71.9 (64.6–77.9)
84	18	1	68.7 (59.1–76.5)
96	9	2	64.9 (52.8–74.7)
120	3	1	44.5 (19.9–66.6)

Note: The survivor functions and 95% confidence intervals (CI) were computed using the Kaplan–Meier method.

**Table 3:** Factors associated with patient survival in peritoneal dialysis, Mexico 2013–2023.

Characteristic	HR (95% CI), *p*	
	Bivariate Analysis	Multiple Analysis
Gender		
Female	1.00	1.00
Male	0.78 (0.48–1.25), 0.300	0.74 (0.45–1.20), 0.218
Age (years)	1.02 (1.01–1.03), 0.014	1.02 (1.01–1.03), 0.040
Treatment modality		
APD	1.00	1.00
CAPD	2.03 (1.18–3.48), 0.010	1.89 (1.08–3.01), 0.026
Year of treatment initiation	1.08 (0.93–1.24), 0.309	1.03 (0.89–1.19), 0.708

Abbreviations: HR, hazard ratio; CI, confidence interval; APD, automated peritoneal dialysis; CAPD, continuous ambulatory peritoneal dialysis. Notes: (1) Cox proportional hazards regression models were used to obtain HRs and 95% CIs. (2) The estimates from the multiple analysis were adjusted for all the variables listed in the table.

## Data Availability

The data presented in this study are available on request from the corresponding author.
